# Supplementary material for: Influence of Tibetan Plateau snow cover on East Asian atmospheric circulation at medium-range time scales
Source: Nat Commun. 2018 Oct 12;9:4243. doi: 10.1038/s41467-018-06762-5 (PMC6185922; doi:10.1038/s41467-018-06762-5)
Supplement: Supplementary file 1 — Supplementary Information [file 41467_2018_6762_MOESM1_ESM.pdf]

Supplementary Information to

Influence of Tibetan Plateau snow cover on East Asian atmospheric circulation at medium-range time scales

Li et al.

Supplementary Fig. 1

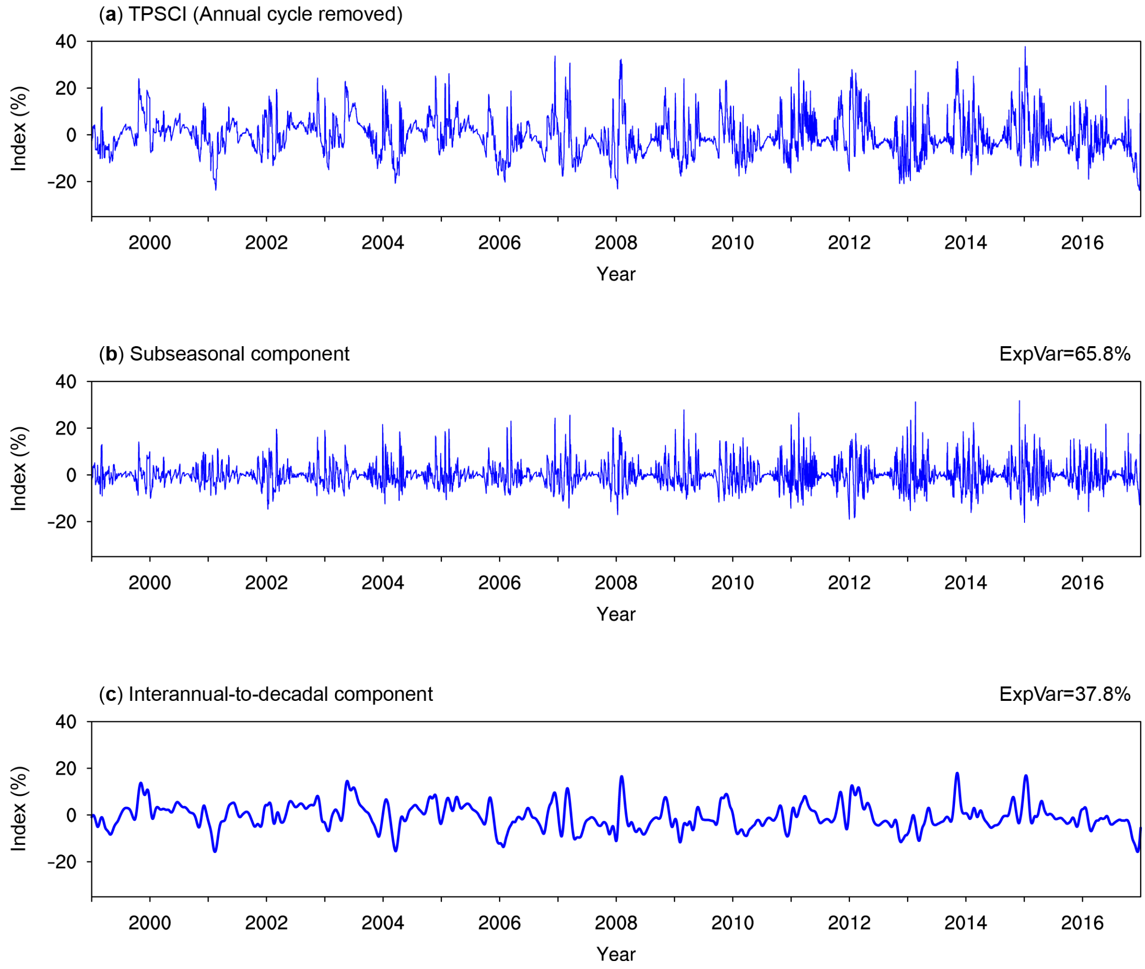

Supplementary Fig. 1 The subseasonal and interannual-to-decadal components of the Tibetan Plateau snow cover index. (**a**) The Tibetan Plateau snow cover index (TPSCI) is defined in the Methods section. Note that we have removed the annual cycle of the raw TPSCI. (**b**) The subseasonal and (**c**) interannual-to-decadal components of the TPSCI. Unit is %. The *x*-axis labels indicate the first day of each year. The two components were subtracted using high-pass and low-pass filters. The cut-off frequencies of both the high-pass and low-pass filters are 1.0/120.0 (days^{-1}). The sum of the time series in (**b**) and (**c**) is the TPSCI. The titles of (**b**) and (**c**) indicate the variance of the TPSCI in (**a**) that each explains. Because the two components are not strictly independent, the sum of the two explained variances is greater than 100%.

Supplementary Fig. 2

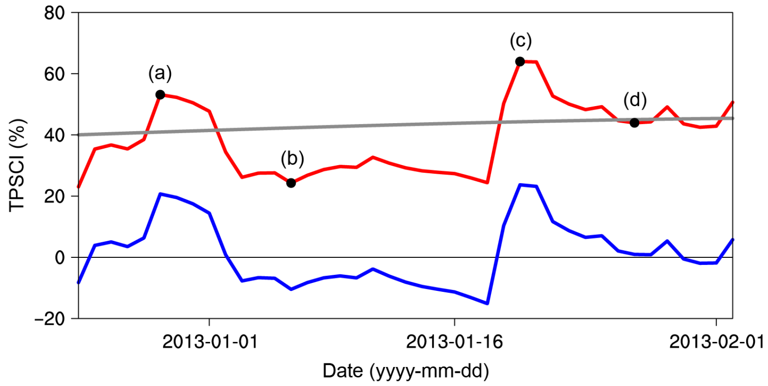

Supplementary Fig. 2 The Tibetan Plateau snow cover index and its subseasonal component in the cold season of 2012/2013. The red line shows the raw Tibetan Plateau snow cover index (TPSCI; percentage of snow-covered area over the Tibetan Plateau). The blue line shows the subseasonal component of the TPSCI. The grey line shows the annual cycle of the TPSCI. The thin black line shows the reference line of zero. The unit is %. (a)−(d) mark the selected dates on which the Tibetan Plateau snow cover spatial distributions are shown in Supplementary Fig. 3.

Supplementary Fig. 3

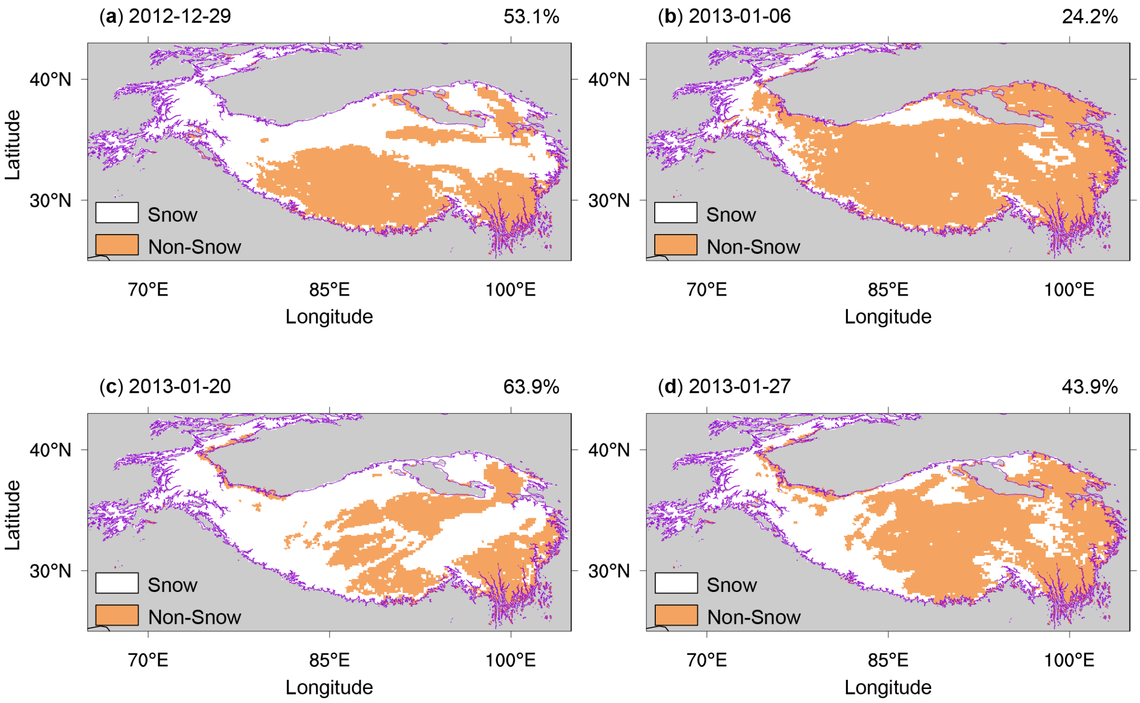

Supplementary Fig. 3 The spatial distribution of Tibetan Plateau snow cover on selected dates. The white area shows the snow-covered grids from the Interactive Multi-Sensor Snow and Ice Mapping System snow cover analysis, while the orange area shows the grids with no snow. The right title in each subfigure represents the percentage of snow-covered area over the Tibetan Plateau. The map of Tibetan Plateau was created by using topographic data from Global Relief Model data of ETOPO1 (doi:10.7289/V5C8276M).

Supplementary Note 1: Explanation for Supplementary Fig. 4

The numerical experiments can reproduce both decreasing (increasing) Tibetan Plateau snow cover index (TPSCI) for positive (negative) anomalous Tibetan Plateau snow cover events within 6 days. The observational TPSCI anomalies fall/rise sharply after 7 days, while the model-simulated TPSCI anomalies still show relatively slow tendencies. Although there is some bias 7 days after the initial date, the numerical experiments can generally reproduce both the persistence of the snow anomaly signal and the decreasing tendency at a medium range.

Supplementary Fig. 4

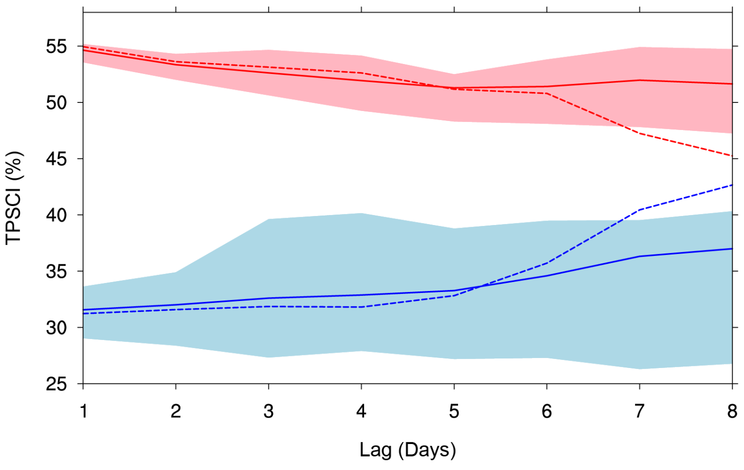

Supplementary Fig. 4 The persistence of the Tibetan Plateau snow cover index. This figure is similar to Figure 4b, but show the results in both the positive Tibetan Plateau snow cover (TPSC) and negative TPSC composites/experiments**.** The *x*-axis represents the number of days lagging the start of each event for the composites or the model initial date. The dashed red line and dashed blue line represent the analysis composites for positive and negative TPSC events, respectively. The solid red line and solid blue line represent numerical experiments of positive and negative anomalous TPSC experiments, respectively. The light shadings represent the range of the TPSC index between the 25th and 75th percentile of the numerical experiment ensembles. The unit is %.

Supplementary Fig. 5

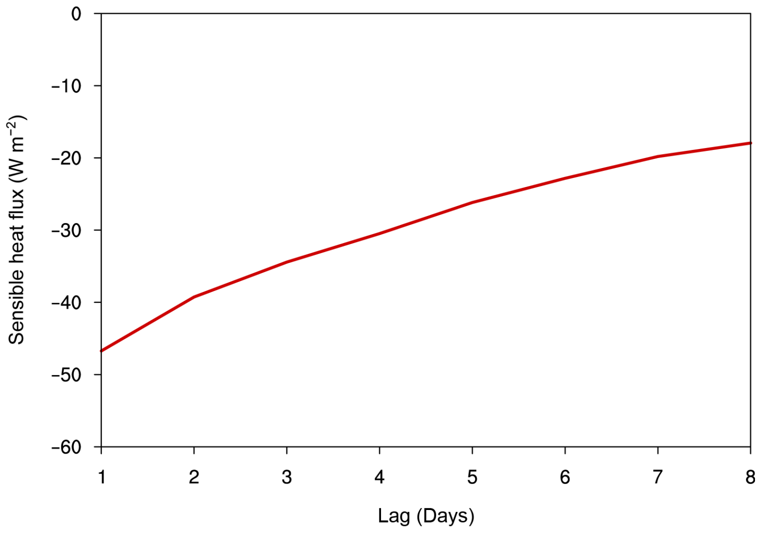

Supplementary Fig. 5 The response of the regionally averaged sensible heat flux over the Tibetan Plateau surface to the subseasonal variability of Tibetan Plateau snow cover in the numerical experiments. The unit is W m^{-2}. The value is the difference between the sensible heat flux of positive and negative anomalous Tibetan Plateau snow cover experiments. The *x*-axis represents the number of lag days from the initial date of the model.

Supplementary Fig. 6

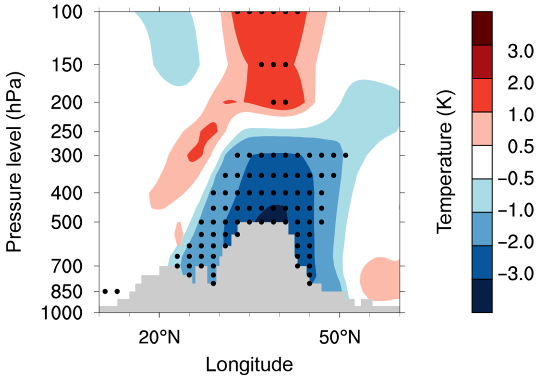

Supplementary Fig. 6 Response of temperature to the subseasonal variability of Tibetan Plateau snow cover in observations. As Fig. 5a, but for the temperature composite in the reanalysis. Stippled regions mark composites with significance at the 99% level (two-side Student's *t* test; see Methods).

Supplementary Fig. 7

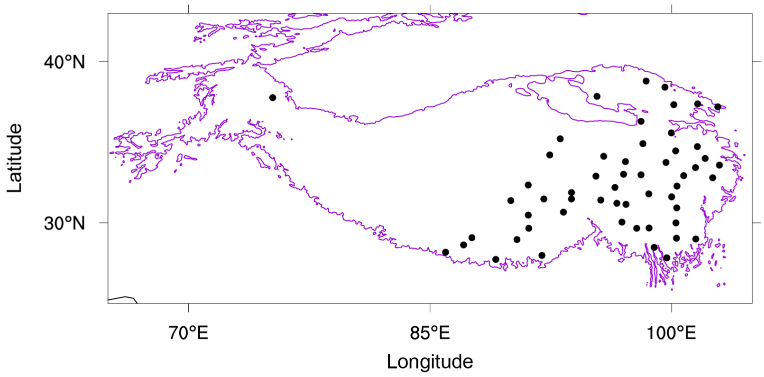

Supplementary Fig. 7 Distribution of meteorological stations over the Tibetan Plateau (black dots). The purple contour marks the regions of the Tibetan Plateau with altitudes higher than 3,000 meters. The map of Tibetan Plateau was created by using topographic data from Global Relief Model data of ETOPO1 (doi:10.7289/V5C8276M).

Supplementary Fig. 8

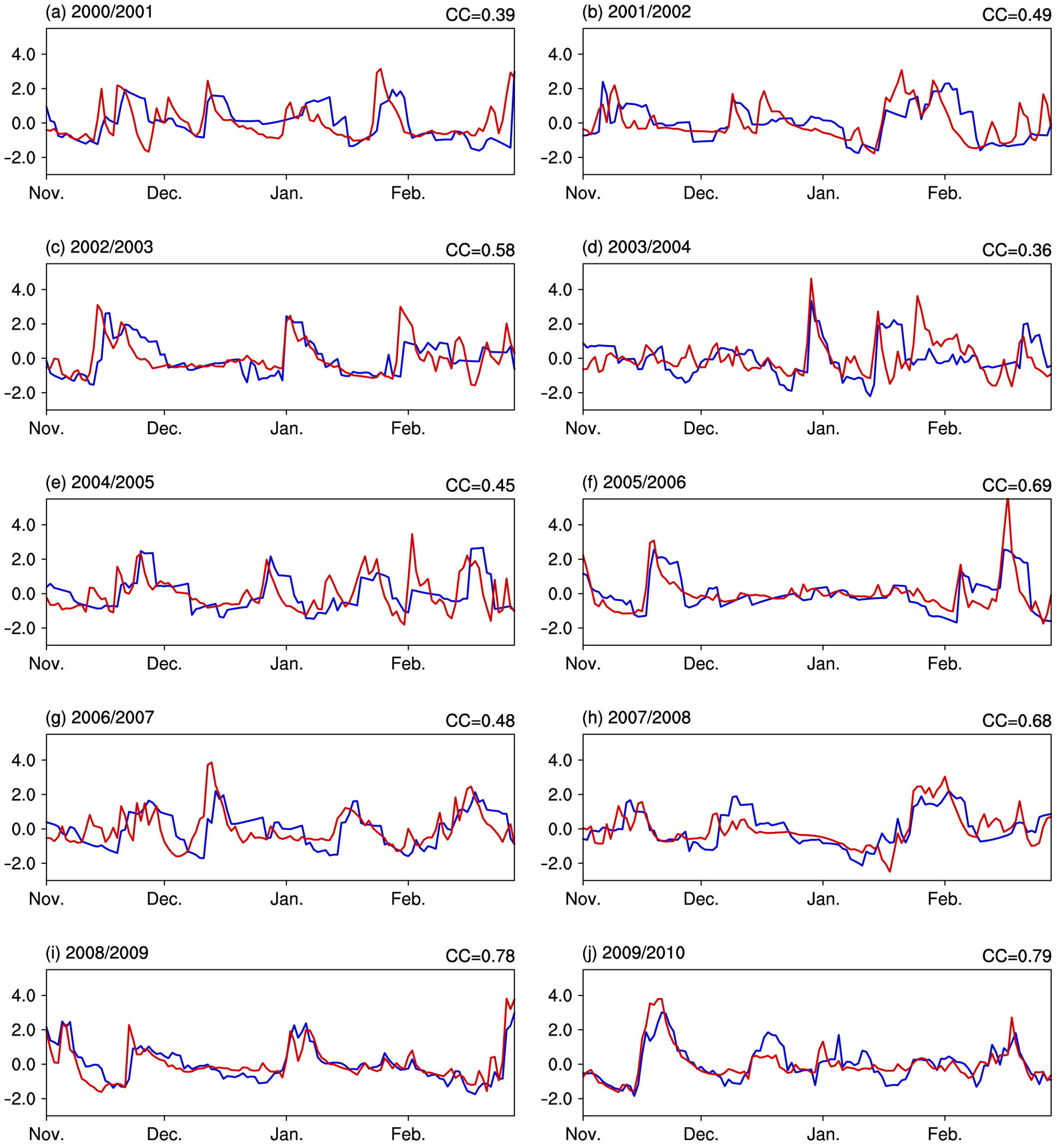

Supplementary Fig. 8 The subseasonal variability of the Tibetan Plateau snow cover index from stations and Interactive Multi-Sensor Snow and Ice Mapping System snow cover analysis. Daily time evolution of the Tibetan Plateau snow cover index for 55 stations (red lines) and Interactive Multi-Sensor Snow and Ice Mapping System snow cover analysis (blue lines). The time series for each winter are standardized. The right title in each plot shows the correlation coefficient (CC) between the two time series for each winter (the sample size $N = 120$). The CC for the two time series for all 10 winters is 0.56 (the sample size $N = 1200$).

Supplementary Fig. 9

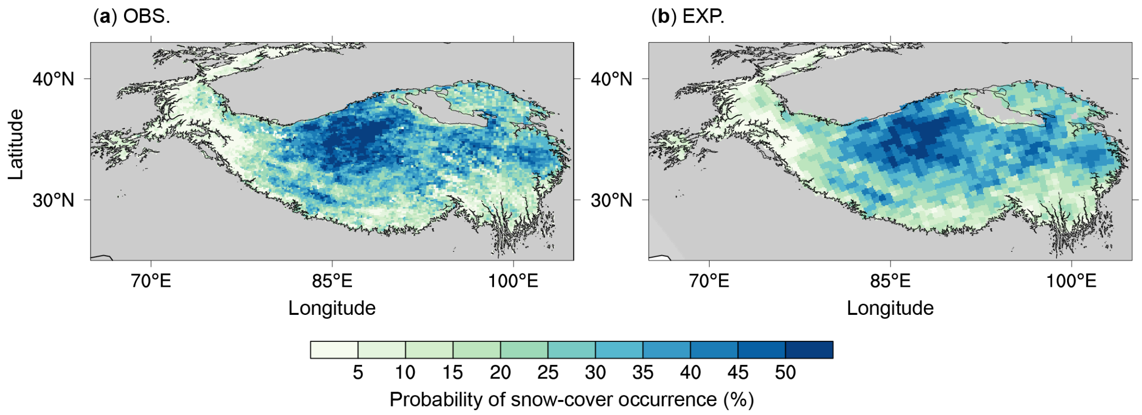

Supplementary Fig. 9 The initial lower boundary condition Tibetan Plateau snow cover based on the Interactive Multi-Sensor Snow and Ice Mapping System snow cover analysis. (**a**) The composites of the daily anomalous TP snow-cover probabilities at a lag of 0 days. (**b**) The difference between the initial snow cover fraction of positive and negative anomalous Tibetan Plateau snow cover experiments (ExpPOS and ExpNEG). The unit is %. Grey areas indicate the areas with altitudes less than 3,000 metres. The map of Tibetan Plateau was created by using topographic data from Global Relief Model data of ETOPO1 (doi:10.7289/V5C8276M).
